# *Boswellia carterii* oleoresin extracts induce caspase-mediated apoptosis and G_1_ cell cycle arrest in human leukaemia subtypes

**DOI:** 10.3389/fphar.2023.1282239

**Published:** 2023-12-14

**Authors:** Matthew Allan Jones, Anna Borun, David James Greensmith

**Affiliations:** Biomedical Research Centre, School of Science, Engineering and Environment, University of Salford, Manchester, United Kingdom

**Keywords:** *Boswellia*, phytomedicine, cancer, leukaemia, apoptosis, cell cycle

## Abstract

**Background:** Leukemias are a common cancer in adults and children. While existing treatments are effective, they are associated with severe side-effects compounded by the emergence of drug resistance. This necessitates the need to develop new drugs and phytopharmaceuticals offer a largely untapped source. Oleoresins produced by plants in the genus *Boswellia* have been used for centuries in traditional medicine and recent work suggests they may exhibit anti-cancer activity. However, the underlying mechanisms remain unclear and most existing research focusses on *Boswellia serrata*; just one of many species in the *Boswellia* genus. To address these limitations, we elucidated the anti-cancer potential and associated mechanisms of action of *Boswellia carterii*.

**Methods:** A methanolic solvent extraction method was optimised. The effect of methanolic extracts of *B. carterii* on leukaemia (K562, MOLT-4 and CCRF-CEM) and normal (PBMC) cell line viability was assessed using MTT assay and flow cytometry. Cell morphology, apoptosis (Annexin-V/propidium iodide), mitochondrial membrane potential (Rhodamine-123) and the cell cycle (propidium iodide) were evaluated using flow cytometry. Regulatory protein expression was quantified using Western Blot.

**Results:** Methanolic extracts of *B. carterii* oleoresin reduced the viability of K562, MOLT-4 and CCRF-CEM cell lines with selectivity indexes of between 1.75 and 2.68. Extracts increased the proportion of cells in late apoptosis by 285.4% ± 51.6%. Mitochondrial membrane potential was decreased by 41% ± 2% and the expression of cleaved caspase-3, -7, and -9 was increased by 5.7, 3.3, and 1.5-fold respectively. Extracts increased the proportion of cells in _sub_G_1_ and G_1_ phase by 867.8% ± 122.9% and 14.0 ± 5.5 and decreased those in S phase and G_2_/M by 63.4% ± 2.0% and 57.6% ± 5.3%. Expression of CDK2, CDK6, cyclin D1, and cyclin D3 were decreased by 2.8, 4.9, 3.9, and 2.5-fold.

**Conclusion:** We are the first to report that methanolic extracts of *B. carterii* are selectively cytotoxic against three leukemia cell lines. Cytotoxic mechanisms likely include activation of the intrinsic apoptotic pathway and cell cycle arrest through downregulation of CDK2, CDK6, cyclin D1, and cyclin D3. Our findings suggest that *B. carterii* may be an important source of novel chemotherapeutic drugs and justifies further investigation.

## 1 Introduction

Leukaemia is a common cancer that presents as increased blood leucocyte count and remains a considerable global health burden (Du et al., 2022). In children and young adults, the incidence of leukaemia increases each year and now accounts for one-third of all cancer diagnoses (Hao et al., 2019; CRUK, 2021). Despite this, improvements in chemotherapeutics—which remain at the forefront of treatment regimens—have produced net 5-year survival rates of between 37% and 89% dependent on leukaemia subtype (Bhayat et al., 2009; Chang et al., 2021; Kantarjian et al., 2021; Sekeres and Taylor, 2022). Drugs commonly used to treat leukaemia include anthracyclines, topoisomerase inhibitors, mitotic inhibitors, alkylating agents and antimetabolites (Estey, 2018) which produce cell cycle arrest and apoptosis via various mechanisms (Ralhan and Kaur, 2007; Luengo et al., 2017; Martins-Teixeira and Carvalho, 2020). However, despite their effectivity against leukaemia, many produce severe short and long-term off-target effects in multiple organ systems (Oeffinger et al., 2006; Armstrong et al., 2014) which are particularly severe in developing children and adolescents (Ikonomidou, 2018). Therefore, there remains a need to develop novel effective chemotherapeutics with fewer off-target effects.

Many anti-cancer drugs are derivatives of plant-based compounds (Demain and Vaishnav, 2011). Yet, phytopharmaceuticals offer a largely untapped source for the development of novel anti-cancer drugs (Mtewa et al., 2021) despite a tendency to exhibit fewer and less severe side-effects (Iten et al., 2002). Plants in the genus *Boswellia* provide a notable example (Lim et al., 2019).

*Boswellia* species are shrub-like trees, widely distributed from the Horn of Africa, throughout the Middle East and into India and China (Abdel-Tawab et al., 2011). These plants produce oleoresins—commonly known as Frankincense—which have been used for centuries as a part of Ayurvedic and traditional medicine practices (Siddiqui, 2011). However, recent evidence suggests that *Boswellia* oleoresins and oleoresin-derived compounds may be effective in the treatment of a variety of diseases. For example, previous work highlights that extracts of *Boswellia* oleoresins show anti-inflammatory (Koeberle et al., 2018) and anti-microbial (Al-Dosary, 2018) activity. Furthermore, several studies show that extracts of the *Boswellia* oleoresins exhibit anti-cancer activity against multiple cancer cell types *in vitro* (Ahmed et al., 2015; Zhang et al., 2016; Mazzio et al., 2017; Ranjbarnejad et al., 2017; Schmiech et al., 2019) through mechanisms such as apoptosis (Moussaieff and Mechoulam, 2009; Ahmed et al., 2015; Ranjbarnejad et al., 2017), and cell cycle arrest (Yazdanpanahi et al., 2014).

However, studies that seek to elucidate the mechanistic actions of *Boswellia* are limited, use a variety of cancer cell types and tend to focus on one particular mechanistic element. As such, a cohesive description of the mechanistic effects of *Boswellia* remains unreported. Furthermore, the majority of existing research focusses on *Boswellia serrata* which is one of many species in the *Boswellia* genus (Moussaieff and Mechoulam, 2009; Abdel-Tawab et al., 2011; Efferth and Oesch, 2022). As a result, much less is known about the biological activity of other accessible *Boswellia* species such as *Boswellia carterii*.

To address these limitations, we sought to investigate the anti-cancer effects of *B*. *carterii* oleoresin extracts against leukemia cell lines. We then took an integrative methodological approach to reveal the associated mechanisms of action thus producing a unified description of the anti-cancer activity of *B*. *carterii*.

## 2 Materials and methods

### 2.1 Chemicals and reagents

All chemicals and reagents were purchased from Fisher scientific, United States, unless otherwise stated.

### 2.2 Phytochemical extraction

The *B. carterii* Birdw oleoresin samples used for this study was provided by United Kingdom Essential oils and authenticated by Steven Holmes as a part of industrial procurement quality control. Voucher specimens are stored at The University of Salford, United Kingdom.

*B. carterii* oleoresin was macerated to a fine powder and then extracted using either acetonitrile (99.5%) (AcN), distilled water (dH_2_O), ethanol (99%) (EtOH), methanol (99.5%) (MeOH) or propan-2-ol (99.5%) (PrOH) at a final concentration of 100 mg/mL. The mixture was then stirred continuously for 2 h at room temperature. After 2 h, the supernatant was removed, and fresh extraction solvent added. This process was repeated three times. The supernatants were pooled then filtered through Whatman no 1 filter paper, before being dried to a powder using a rotary evaporator (Eppendorf, United States) and stored at −20°C. All extracts used for further experimentation were dissolved in dimethyl sulphoxide (DMSO) to a stock concentration of 50 mg/mL.

Each of the extracts is characterized by gradient high-performance liquid chromatography with diode-array detection (HPLC-DAD) as described in Supplementary Data S1.1 with spectra for each extraction method shown in Supplementary Figures S1–S4.

### 2.3 Cell culture

Three human leukaemia cell lines were used; K562, a chronic myeloid leukaemia (CML) and MOLT-4 and CCRF-CEM, which are both acute lymphoblastic leukaemias (ALL). Cell lines were provided by the Kidscan Children’s cancer research laboratories at The University of Salford, United Kingdom and originally sourced from American Type Culture Collection (ATCC, United States). All 3 cell lines were cultured using Roswell Park Memorial Institute (RPMI) 1,640 cell culture medium (Gibco, United Kingdom) supplemented with 10% *v/v* foetal bovine serum (FBS) (Gibco, United Kingdom) and 1% *v/v* penicillin-streptomycin (Penicillin: 10,000 units/mL, Streptomycin: 10,000 μg/mL) (Gibco, United Kingdom) as described in Gaigneaux et al. (2002) and Deesrisak et al. (2021). All cells were maintained at 37°C in a 5% CO_2_ atmosphere.

### 2.4 Peripheral blood mononuclear cell isolation

Peripheral blood mononuclear cells (PBMC’s) were isolated, to serve as a normal cell type, from Type O positive whole human blood (National Health Service Blood & Transplant, Manchester, United Kingdom) as described by Chen et al. (2020). Following isolation, PBMC’s were maintained in RPMI 1640 supplemented with 10% FBS and 1% penicillin-streptomycin at 37°C in a 5% CO_2_ atmosphere.

### 2.5 Measurement of cytotoxicity and calculation of anti-cancer selectivity

The cytotoxic effects of the *B. carterii* oleoresin extracts were determined using 3-(4,5-dimethylthiazol-2-yl)-2,5-diphenyltetrazolium bromide (MTT) assays, as described by Carreño et al. (2021) against K562, MOLT-4, CEM-CCRF and PBMC’s. To evaluate the time dependent effects of the *B. carterii* oleoresin extracts, MTT assays were performed at 24, 72, and 120 h. For all experiments, 50 µM doxorubicin hydrochloride was used as a positive control. IC_50_ values were calculated using SigmaPlot 12.3 (Systat Software, Inc., United States). The selectivity of the *B. carterii* oleoresin methanolic extract was calculated using the equation shown below.

$$Selectivity=\frac{IC50\;non-cancer\;cells}{IC50\;cancer\;cells}$$

### 2.6 Evaluation of cellular morphology

Cellular morphology was measured at a constant temperature of 37°C in a 5% CO_2_ atmosphere by time-lapse microscopy using a Cytation 3 cell Imaging multi-mode reader (BioTek Instruments, United States). Leukaemia cells were imaged hourly for 72 h, at ×100 magnification, following exposure to 25–100 μg/ml *B. carterii* oleoresin methanolic extract. Key morphological changes were examined and annotated offline using ImageJ (National Institutes of Health, United States) (Schneider et al., 2012).

### 2.7 Evaluation of cell size and granularity

Cell densities of 50,000 cells were treated for 24–72 h with 25–100 μg/ml *B. carterii* oleoresin methanolic extract then washed with 1x PBS. A minimum of 10,000 cells were measured using flow cytometry (FACSverse flow cytometer & FACSuite software, Becton, Dickinson and Company, United States) to determine relative cell size (using forward scatter) and granularity (side scatter) as described in Haynes et al. (2009). All changes were normalised to treatment with the vehicle (0.5% DMSO *v/v*) alone.

### 2.8 Evaluation of apoptosis by annexin-V and propidium iodide co-staining

Cell densities of 100,000 cells were incubated for 48 h with 25–100 μg/ml *B. carterii* oleoresin methanolic extract or a vehicle control (0.5% *v/v* DMSO) then washed with 1x PBS. Cells were then co-stained with annexin-V conjugated to FITC (to measure apoptosis) and propidium iodide (to measure necrosis) using a proprietary kit (Invitrogen, United States) as per manufacturers instructions. Annexin-V (Excitation: 490, Emission: 525 nm) and propidium iodide (Excitation: 535, Emission: 617 nm) fluorescence in a minimum of 10,000 cells was measured using flow cytometry (FACSverse flow cytometer & FACSuite software).

### 2.9 Qualitative assessment of mitochondrial membrane potential

The effect of the *B. carterii* oleoresin methanolic extract on leukaemia mitochondrial membrane potential (Δψ_m_) was evaluated by flow cytometry as previously described by Panwar et al. (2020). Cell densities of 100,000 cells were incubated for 24–72 h with 25–100 μg/ml *B. carterii* oleoresin extract, a vehicle control (0.5% *v/v* DMSO) or a positive control (the oxidative phosphorylation uncoupler carbonyl cyanide 3-chlorophenylhydrazone (CCCP) (Sigma Aldrich, United States). Cells were then washed with 1x PBS and loaded with 200 nM rhodamine-123. Rhodamine-123 was excited at 508 nm and fluorescence measured at 528 nm using a FACSverse flow cytometer & FACSuite software.

### 2.10 Cell cycle analysis

To assess the effect of the *B. carterii* methanolic extract on the cell cycle, was conducted as previously described by Pozarowski and Darzynkiewicz (2004). Cell densities of 200,000 cells were incubated for 24 or 48 h with 25–100 μg/ml *B. carterii* oleoresin extract, a vehicle control (0.5% *v/v* DMSO). Cells were then washed with 1x PBS and fixed with ice-cold 70% *v/v* ethanol. Following fixation cells were treated with 100 μg/mL RNase A (ThermoFisher Scientific, United States) and stained with 50 μg/mL propidium iodide (Excitation: 535, Emission: 617 nm) [(Acros Organics, United States)]. A minimum of 10,000 cells were then immediately analysed using a FACSverse flow cytometer & FACSuite software.

### 2.11 Quantification of regulatory proteins using Western blotting

Protein was extracted from 1 × 10^6^ cells pre-treated with varying concentrations of the *B. carterii* oleoresin methanolic extract for 48 h using radioimmunoprecipitation assay lysis buffer (Thermo Fisher Scientific, United States). Protein was quantified using bicinchoninic acid protein assays. A concentration of 50 µg of whole cell protein lysate for each condition was separated using sodium dodecyl sulphate (SDS) PAGE over a gradient of 4%–20% (Merck, United States) and transferred to 0.45 µm Polyvinylidene fluoride (PVDF) membranes (Immobilon, United States) as described in Mahmood and Yang (2012). The primary and secondary antibodies utilised are described within the Supplementary Section S1.2. Protein expression was then visualised by chemiluminescence using Crescendo Western horseradish peroxidase (HRP) substrate (Immobilon, United States). Chemiluminescent Images were taken using a G:Box Chemi-XX6 imager and the GeneSys V1.5.2.0 software (both Syngene, United States). Protein abundance was quantified using the software package ImageJ (National Institutes of Health, United States).

### 2.12 Statistical analysis

Appropriate statistical tests (as indicated in figure legends) were performed using SigmaPlot 12.3 (Systat Software, Inc., United States). Statistical significance was accepted when *p* < 0.05. A minimum of three independent repeats were conducted for all experiments.

### 2.13 Ethical approval

Ethical approval was granted (STR1718-35) by the University of Salford Research, Innovation and Academic Engagement Ethical Approval Panel.

## 3 Results

### 3.1 The effects of extraction solvent on *B. carterii* oleoresin extract yield

Supplementary Figure S5A shows the extraction yields produced by acetonitrile, distilled water, ethanol, methanol, and propanol. Statistical indicators have been removed for clarity, though Supplementary Figure S5B provides *p*-values for all pairwise comparisons. The yields produced by ethanol, methanol and propanol were 50 ± 7, 55% ± 3% and 50% ± 8% respectively (*n* = 3) and significantly greater than those produced by acetonitrile and water; 14% ± 5% and 8% ± 2% respectively (*n* = 3, *p* < 0.001). The yield produced between each alcoholic and non-alcoholic solvent did not differ significantly.

### 3.2 The concentration-dependent effects *B. carterii* oleoresin extract on cytotoxicity

**FIGURE 1:**
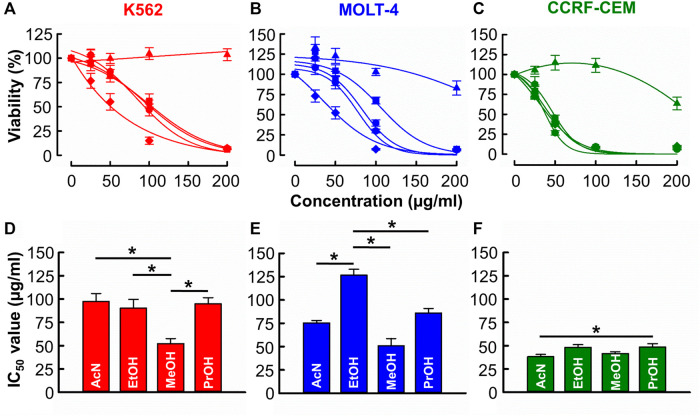
The of effects *B. carterii* oleoresin solvent extracts on leukemia cytotoxicity. **(A–C)** Dose response curves comparing the cytotoxicity of *B. carterii* oleoresin solvent extracts [Acetonitrile (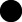), distilled water (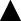), Ethanol (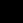), Methanol (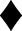), Propan-2-ol (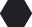)] in K562, MOLT-4 and CCRF-CCM. In all examples, cells were treated for 72 h and all data points are normalised to vehicle alone (0.5% *v/v* DMSO). **(D–F)** Corresponding normalsied mean ± SEM IC_50_ values from 12 repeats. Statistically significant (*p* < 0.05) pairwise comparisons are indicated by asterisks (*).

Figures 1A–C shows that with the exception of distilled water, all solvents produced a concentration-dependent cytotoxic effect against CML (K562) and ALL (MOLT-4 & CCRF-CEM) cell lines. Figures 1D–F show the derived IC_50_ values. In K562 cells (Figure 1D), the IC_50_ of methanol (52.2 ± 5.5 μg/mL) was significantly less than those of acetonitrile, ethanol, and propanol (97.5 ± 8.6 μg/mL, 90.3 ± 9.3 μg/mL, 95.0 ± 6.5 μg/mL respectively, *n* = 12, *p* < 0.001). In MOLT-4 cells (Figure 1E), the IC_50_ of acetonitrile, methanol, and propanol (75.5 ± 2.7, 51.1 ± 7.7 and 86.3 ± 4.8 μg/mL respectively) was significantly less than that of ethanol (126.8 ± 6.2 μg/mL) though the IC_50_ of methanol was significantly lowest (*n* = 12, *p* < 0.001). In CCRF-CEM cells (Figure 1F) IC_50_ values for acetonitrile (38.3 ± 2.5 μg/mL), ethanol (48.3 ± 2.9 μg/mL), methanol (41.6 ± 1.9 μg/mL) and propanol (48.6 ± 3.5 μg/mL) were similar with the only significant difference being that acetonitrile produced a lower IC_50_ than propanol (*n* = 12, *p* = 0.032).

### 3.3 The time-dependence and specificity of *B. carterii* oleoresin methanolic extract cytotoxicity

**FIGURE 2:**
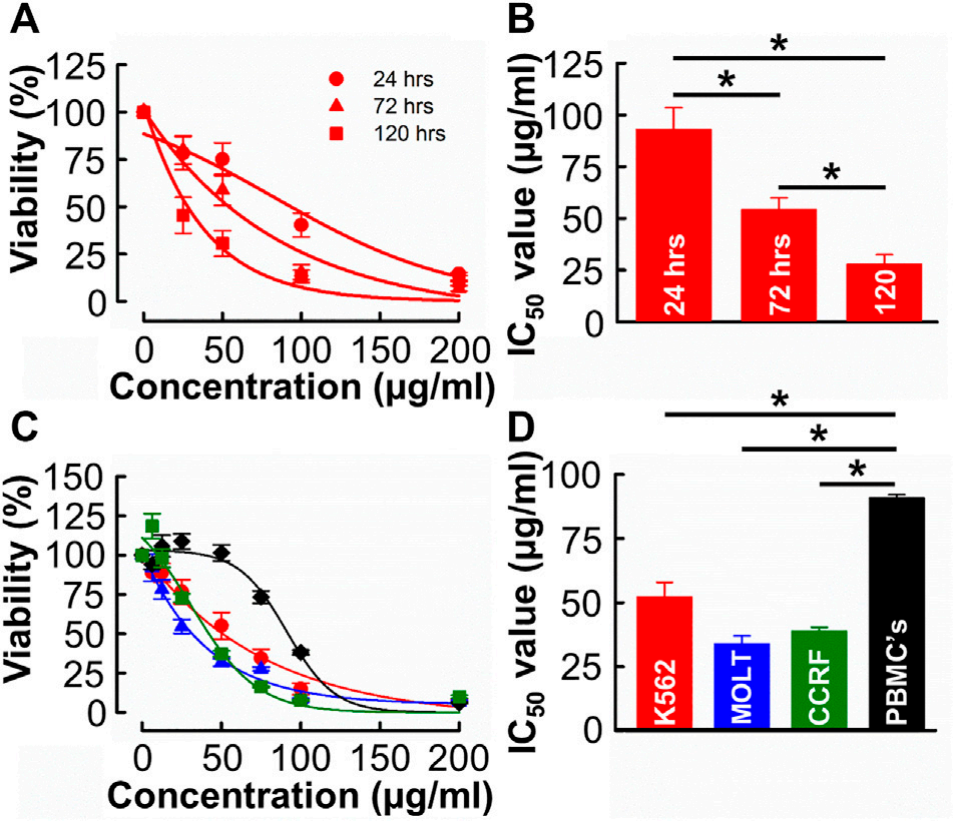
The time and concentration dependent effects of *B. carterii* oleoresin methanolic extract on leukemia and peripheral blood mononuclear cell cytotoxicity **(A)** Dose response curves comparing the cytotoxicity of *B. carterii* methanolic extract following treatment for 24, 72 and 120 h. In all examples, K562 cells were used. All data points are normalised to vehicle alone (0.5% *v/v* DMSO). **(B)** Corresponding normalised mean ± SEM IC_50_ values from 12 repeats. **(C)** Dose response curves comparing concentration dependent cytotoxicity of the *B. carterii* oleoresin methanolic extract against K562 (red), MOLT-4 (blue) and CCRF-CEM (green) leukaemia cell lines and the non-cancerous PBMC’s (black). In all examples, cells were treated for 72 h, and all data points are normalised to vehicle alone (0.5% *v/v* DMSO). **(D)** Corresponding normalized mean ± SEM IC_50_ values from 12 repeats. Statistically significant (*p* < 0.05) pairwise comparisons are indicated by asterisks (*).

Given the relative yield and potency values, methanolic extracts were used for all subsequent experiments (see Section 4.1 for detailed rationale). The independent experiment shown in Figure 2A was designed to evaluate the time-dependent effect of *B. carterii* in K562 cells. The effect of each concentration tested on viability increases with incubation time. This is quantified in Figure 2B which compares the IC_50_ values of equivalent dose response curves following 24-, 72- and 120-h incubation periods. This reveals a significant time-dependent effect (24 h; 92.8 ± 10.6, 72 h; 54.2 ± 5.7, 120 h; 27.9 ± 4.6 μg/mL, *n* = 12, *p* < 0.001). This pattern of effect was also observed in MOLT-4 and CCRF-CEM cells (Supplementary Figure S6A, D).

We next evaluated the specificity of effect of *B. carterii* by comparing dose response curves in leukemia cells to that in an equivalent normal cell-line (PBMCs, Figure 2C). The average IC_50_ value (Figure 2D) in PBMC cells (90.8 ± 1.3 μg/mL) was significantly greater than those in K562, MOLT-4 and CCRF cells (52.08 ± 5.9, 33.8 ± 3.2, 38.7 ± 1.7 μg/mL respectively, *n* = 12, *p* < 0.001). The calculated selectivity indices towards K562, MOLT 4 and CCRF-CEM were 1.75, 2.68, and 2.39 respectively.

### 3.4 The effect of *B. carterii* oleoresin methanolic extract on cell morphology

**FIGURE 3:**
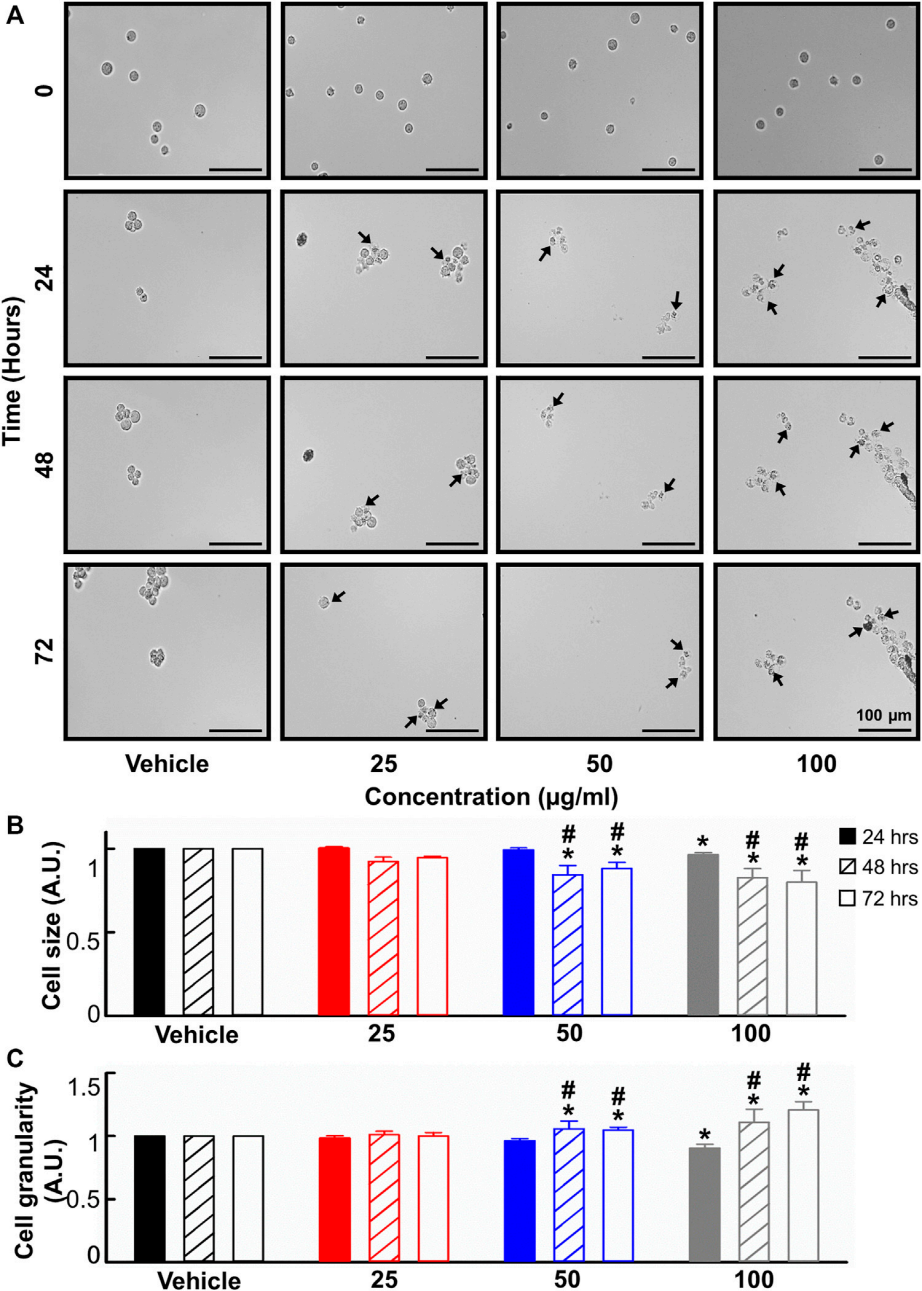
The effects of *B. carterii* oleoresin methanolic extract on cell morphology. **(A)** Specimen micrographs showing K562 morphology following treatment with *B. carterii* oleoresin methanolic extract for 24, 48, and 72 h. Arrows indicate examples cells with key morphological changes. Scale bars represent 100 µm. **(B)** Normalised mean ± SEM cell size. **(C)** Normalised mean ± SEM cell granularity. Means are calculated from 6 repeats. Asterisks (*) indicate concentration means that are statistically different to vehicle alone (0.5% *v/v* DMSO). Hashes (#) indicate incubation-time means that are statistically different to 24 h for a given concentration.

Given the anti-leukemic activity of the *B. carterii* oleoresin, its effect on cell morphology was evaluated. A time and concentration-dependent decrease in cell size and increase in cell granularity is apparent upon visual inspection of the live-cell, time-lapse micrographs shown in Figure 3A. These phenomena were quantified using flow cytometry. Figures 3B, C show that following a 24-h incubation, 100 μg/ml *B. carterii* oleoresin produced a modest 3.6% ± 1.1% decrease (*n* = 6, *p* = 0.03) and 9.6% ± 3.2% decrease (*n* = 6, *p* = 0.007) in cell size and granularity respectively. For both, a time-dependence of effect was observed whereby following 48 and 72-h incubation periods, 50 μg/ml *B. carterii* oleoresin also significantly altered cell morphology by 1) decreasing cell size while 2) increasing granularity. For example, following a 72-h incubation, *B. carterii* oleoresin produced a 20.1% ± 7.1% decrease (*n* = 6, *p* = 0.005) and 20.6% ± 6.6% increase (*n* = 6, *p* = 0.003) in cell size and granularity respectively.

### 3.5 The effects of *B. carterii* oleoresin methanolic extract on apoptosis and necrosis

**FIGURE 4:**
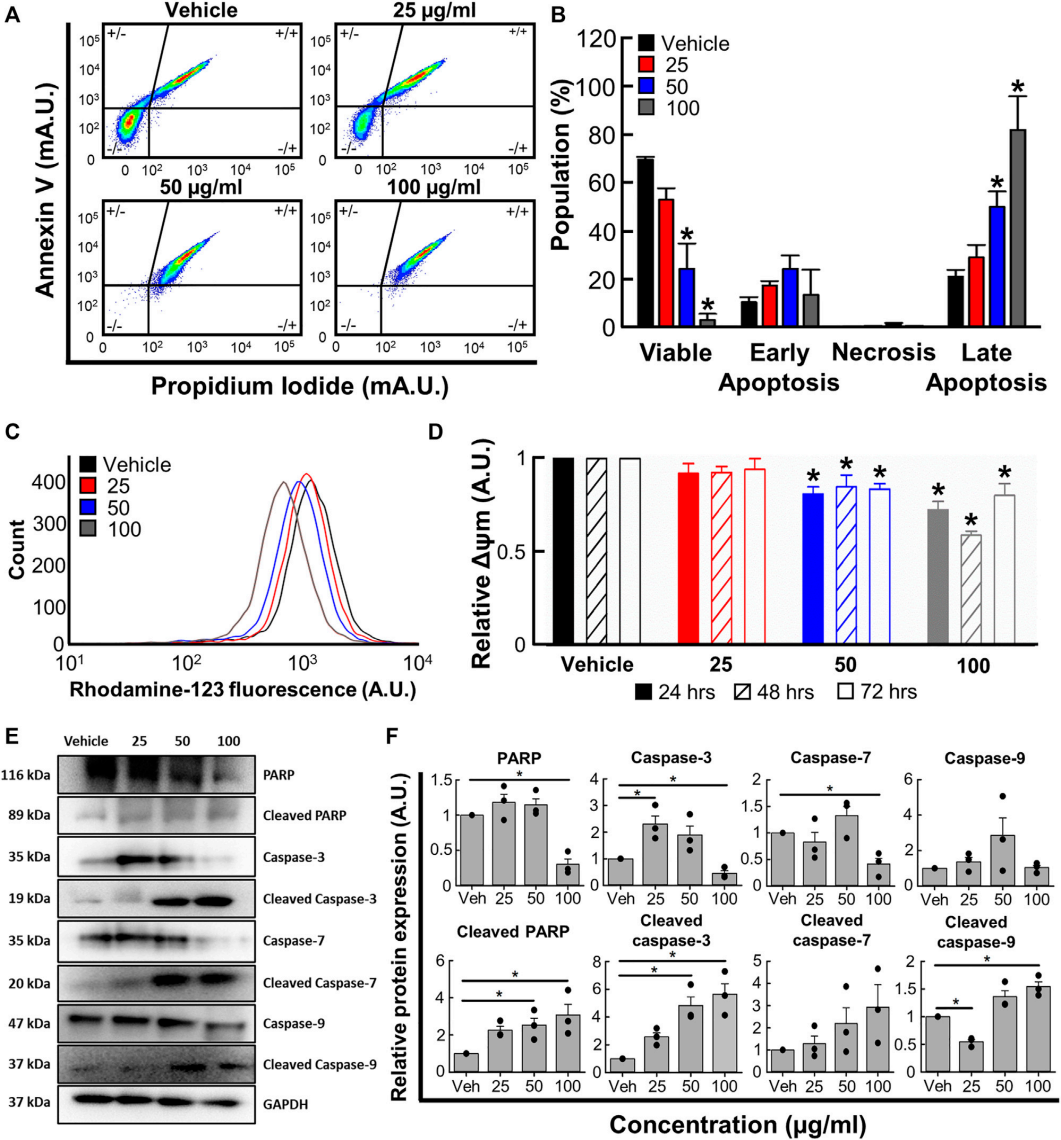
The effects of *B. carterii* oleoresin methanolic extract on apoptosis. **(A)** Specimen flow cytometry plots showing K562 annexin V/propidium iodide co-staining following treatment with *B. carterii* oleoresin methanolic extract for 48 h **(B)** Normalised mean ± SEM cell state proportion from 4 repeats. **(C)** Specimen flow cytometry plot showing the effect of *B. carterii* oleoresin methanolic extract on rhodamine-123 fluorescence in K562 cells. **(D)** Normalised mean ± SEM mitochondrial membrane potential (Δψ_m_) following treatment for 24, 48, and 72 h. Mean calculated from 6 repeats. **(E)** Specimen Western blots for key apoptosis regulatory proteins following treatment for 48 h. GAPDH served as a loading control. **(F)** Relative mean ± SEM protein expression of caspases-3, -7, -9, PARP and their associated cleavage fragments in K562 cells following treatment for 48 h. All means calculated from 3 repeats. Asterisks (*) indicate concentration means that are statistically different to vehicle alone (0.5% *v/v* DMSO).

To determine whether the changes reported in 3.4 were associated with apoptosis, we next used flow cytometry with Annexin V and propidium iodide co-staining. Figures 4A, B show that *B. carterii* oleoresin produced a concentration dependent decrease in cell viability and increase in late apoptosis. For example, at the highest concentration tested (100 μg/mL) *B. carterii* oleoresin decreased the proportion of viable cells by 95.1% ± 3.8% (Vehicle; 69.4% ± 1.2%, 100 μg/mL; 3.3% ± 2.5%, *n* = 4, *p* < 0.001) and increased the proportion of cells in late apoptosis by 285.4% ± 51.6% (Vehicle; 21.1% ± 2.8%, 100 μg/mL; 81.7% ± 13.8%, *n* = 6, *p* < 0.001). Over the time-course of our experiments, *B. carterii* oleoresin did not induce early apoptosis or necrosis.

We next evaluated the effect of *B. carterii* oleoresin on mitochondrial membrane potential (Δψ_m_) by measuring rhodamine-123 fluorescence with flow cytometry. The specimen flow plot (Figure 4C) shows a leftward shift in rhodamine-123 fluorescence indicating a decrease in mitochondrial membrane potential. The mean data shown in Figure 4D demonstrates this effect is concentration, but not incubation time dependent. For example, incubation with the highest concentration tested (100 μg/mL) for an intermediate period (48 h) decreased relative mitochondrial membrane potential by 41% ± 2% (*n* = 6, *p* < 0.001).

Western blotting revealed significant modifications to pro-apoptotic protein expression following treatment for 48 h (Figures 4E, F). For example, 100 μg/ml *B. carterii* oleoresin decreased relative PARP expression by 3.3-fold (*n* = 3, *p* = 0.003) while the 89 kDa cleavage fragment of PARP was increased 3.1-fold (*n* = 3, *p* = 0.023). The concentration-dependent effect of *B. carterii* oleoresin on caspase-3 and 7 expression was complex, though 100 μg/mL reduced expression by 2.2 (*n* = 3, *p* = 0.01) and 2.4 (*n* = 3, *p* = 0.01) fold respectively. Caspase 9 expression was unaltered. *B. carterii* oleoresin produced a concentration-dependent increase in cleaved caspase-3, cleaved caspase-7 and cleaved caspase-9 expression with 100 μg/mL producing a 5.7 (*n* = 3, *p* = 0.003), 3.3 (*n* = 3, *p* = 0.03) and 1.5 (*n* = 3, *p* = 0.01) fold increase respectively.

### 3.6 The effects of *B. carterii* oleoresin methanolic extract on cell cycle regulation

**FIGURE 5:**
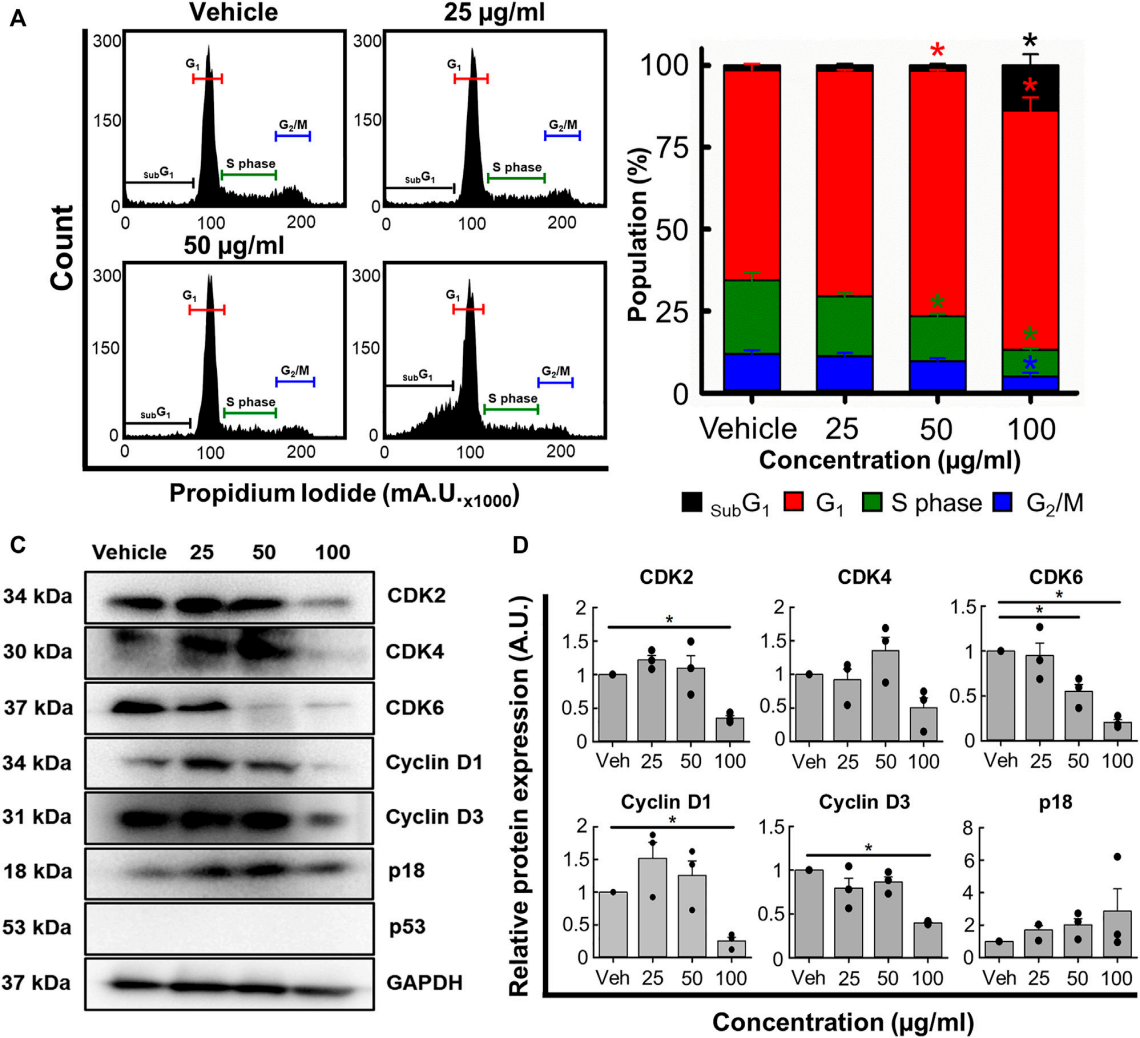
The effects of the *B. carterii* oleoresin methanolic extract on cell cycle regulation. **(A)** Specimen flow cytometry plots showing the effect of *B. carterii* oleoresin methanolic extract on cell cycle regulation following treatment for 48 h. **(B)** Normalised mean ± SEM cell cycle phase proportionality. Means calculated from 4 repeats. **(C)** Specimen Western blots for key cell cycle regulatory proteins following treatment for 48 h. GAPDH served as a loading control. **(D)** Relative mean ± SEM protein expression of CDK2, CDK4, CDK6, cyclin D1, cyclin D3 and p18 following treatment for 48 h. All means calculated from 3 repeats. Asterisks (*) indicate concentration means that are statistically different (*p* < 0.05) to vehicle alone (0.5% *v/v* DMSO).

Next, we used flow cytometry to determine if *B. carterii* oleoresin altered cell cycle regulation. Figure 5A show specimen cell cycle plots. Associated mean data (Figure 5B) reveals a concentration-dependent effect on the proportion of cells in sub G_1_, G_1_, S phase and G_2_. 100 μg/mL increased the proportion of cells in sub G_1_ and G_1_ by 867.8% ± 122.9% (*n* = 4, *p* < 0.001) and 14.0% ± 5.5% (*n* = 4, *p* = 0.013) respectively. The proportion of cells in S phase and G_2_ were decreased by 63.4% ± 2.0% (*n* = 4, *p* < 0.001) and 57.6% ± 5.3% (*n* = 4, *p* = 0.001) respectively.

To explain these changes, we next examined expression of key cell cycle regulatory proteins (Figures 5C, D). 100 μg/ml *B. carterii* oleoresin significantly decreased CDK2, CDK6, cyclin D1 and cyclin D3 expression by 2.8-fold (*n* = 3, *p* = 0.025), 4.9-fold (*n* = 3, *p* = 0.002), 3.9-fold (*n* = 3, *p* < 0.001) and 2.5-fold (*n* = 3, *p* = 0.003) respectively. CDK 4 was decreased by 2-fold but did not reach significance (*n* = 3, *p* = 0.059). The expression of p18 was unaltered.

## 4 Discussion

Over the last 40 years, the development of effective and specific treatments means the prognosis and long-term survival rate for leukaemia has improved considerably. However, these trends may be compromised by the emergence and increased incidence of chemotherapeutic resistant leukaemia’s (Zhang et al., 2019; Niu et al., 2022; Poudel et al., 2022). Furthermore, it remains the case that many existing chemotherapeutic strategies are associated and the severe side-effects, which is especially the case in children. For both reasons, the discovery and development of novel chemotherapeutics remains a priority.

Natural products are an important source given 1) the richness and diversity of potentially therapeutic species, many of which remain unexplored and 2) that compounds isolated from natural sources frequently produce fewer or attenuated side-effects (Cragg and Newman, 2005). One plant genus of interest is *Boswellia*, which has been used in traditional Chinese and Indian medicine for centuries. More recently, several studies show *B. serrata* to be an effective anti-cancer chemotherapeutic *in vitro* (Jaafari-Ashkavandi et al., 2017; Ranjbarnejad et al., 2017; Alipanah and Zareian, 2018; Schmiech et al., 2019). However, a cohesive description of the mechanistic effects of *Boswellia* remains unreported. Furthermore, the majority of existing research focusses on *B. serrata* which is one of many species in the *Boswellia* genus. To address these limitations, we used an integrated approach to elucidate the anti-leukemic potential and mechanisms of effect of *B. carterii.*

### 4.1 Which extraction solvent produces the greatest yield and cytotoxic potential?

Previous investigators predominantly use alcoholic extractions to evaluate the biological activity of *Boswellia* oleoresins (Uma Mahesh et al., 2013; Ahmed et al., 2015; Zhang et al., 2016; Alipanah and Zareian, 2018). However, it wasn’t clear if alcoholic solvents are optimal given that no previous studies have directly compared the extraction yield and biological activity of alternative solvent extracts. Our findings revealed that alcohol-based solvents 1) produce the highest yield (Supplementary Figure S5) and 2) appear to effectively isolate potentially biologically active compounds (Figure 1). Given the high proportion of alcohol soluble material within *Boswellia* oleoresins (Abdel-Tawab et al., 2011), this observation may not surprise. However, we deemed it important to rule out the potential superiority of non-alcoholic solvents.

Integrating the data of Supplementary Figure S5 and 1 reveals that of the alcohols tested, methanol produces the highest yield and extracts with the greatest potency and diversity of effect against leukemia cell lines. For this reason, methanolic extracts were used in all further experiments. Methanol is a commonly used as a *Boswellia* extraction solvent (Yazdanpanahi et al., 2014; Ranjbarnejad et al., 2017), though our study is the first to empirically demonstrate it is indeed most effective.

### 4.2 The cytotoxic profile of *B. carterii*

*B. carterii* oleoresin methanolic extract was cytotoxic against CML (K562) and ALL (MOLT-4 and CCRF-CEM) leukaemia in a time (Figures 2A, B) and concentration (Figures 2C, D) manner. This contributes to a more complete understanding of the *B. carterii* cytotoxic profile, and we are the first to report cytotoxic effects in leukemia.

Clinically used chemotherapeutic agents are associated with severe and long-term side effects (Pearce et al., 2017; Nurgali et al., 2018). While it is encouraging that clinical trials using other *Boswellia* species report few off target effects (Sengupta et al., 2008), similar evidence does not exist for *B. carterii* specifically. As such, we also began to investigate the anti-cancer selectivity of the *B. carterii* oleoresin methanolic extract. Figures 2C, D show minimal cytotoxic activity against human PBMC’s over anti-leukemic concentration ranges. Here, the IC_50_ value of 90.8 ± 1.3 μg/mL was around 2–3 times that against K562, MOLT-4 and CCRF cell lines. Measured IC_50_ values were used to calculate selectivity indexes. This is important, as selectivity indexes calculated during *in vitro* studies predict the likelihood of side effects when compounds are administered *in vivo* (Peña-Morán et al., 2016). The selectivity indexes for K562, MOLT-4 and CCRF-CEM were 1.75, 2.68 and 2.39 respectively which are considered mildly selective according to the recognised standard scale (Peña-Morán et al., 2016). However, it should be noted that the methanolic extract of *B. carterii* oleoresin is *more* selective than clinically employed chemotherapeutic agents such as anthracyclines, which have selectivity indexes in the region of 0.2–1.5 (Badmus et al., 2019).

### 4.3 Does the methanolic extract of *B. carterii* produce apoptosis or necrosis?

Previous studies show oleoresin extracts from other *Boswellia* species induce apoptotic cell death in colorectal (Ahmed et al., 2015; Ranjbarnejad et al., 2017) breast (Yazdanpanahi et al., 2014) and oral squamous cell (Jaafari-Ashkavandi et al., 2017) carcinomas. However, the precise pathways and molecular mechanisms remain ambiguous.

Visual review (Figure 3A) of cells revealed concentration and time-dependent morphological changes. These included increased granularity (including formation for apoptotic blebs), decreased cell size and evidence of pyknosis The effects on granularity and cells size were confirmed quantitively using flow cytometry (Figures 3B, C) and are consistent with apoptosis (Elmore, 2007; Jiang et al., 2016). Further evidence for a pro-apoptotic action is provided by annexin V/propidium iodide co-staining (Figure 4A) thus molecular investigation of flippase phosphatidylserine translocation; a phenomenon commonly associated with apoptosis (Segawa and Nagata, 2015). The mean data shown in Figure 4B 1) confirms the effect on cell viability reported in Figure 2 and 2) reveals that apoptosis but not necrosis is responsible.

Our next experiments sought to establish whether apoptosis resulted from activation of the intrinsic or extrinsic pathway. Figures 4C, D show that *B. carterii* oleoresin methanolic extract decreases mitochondrial membrane potential. In apoptotic cells, mitochondrial membrane potential is decreased by the BCL-2 family of proteins (Bid, Bax, and Bak), which form oligomers on mitochondrial outer membrane. This process results in membrane permeabilisation so the release of intramitochondrial factors that include cytochrome C (Wang and Youle, 2009; Jin et al., 2010). This mitochondrial deplolarisation is a key factor in the development of the apoptosome in conjunction with caspase 9 and more strongly associated with the intrinsic pathway (Peña-Blanco and García-Sáez, 2018; Al-Aamri et al., 2021).

Western blotting revealed increased cleavage of the DNA repair enzyme PARP, the loss of which is associated with caspase activation to prevent depletion of adenosine triphosphate pools (Chaitanya et al., 2010). Furthermore, we observed increased cleavage of the cysteinyl proteases caspases-3, -7 and 9 (Figures 4E, F). Cleavage of caspase-3 and -7 coverts them to their active isoforms; a process vital for the effector pathways of apoptosis (Li and Yuan, 2008; Li et al., 2017). That we see an upregulation of cleaved caspase-9 strongly suggests it is activation of the *intrinsic* pathway that leads to activation of effector pathway (Li et al., 2017).

The induction of apoptosis without necrosis is an important consideration for prospective cytotoxic agents. Cells killed via apoptosis can be removed by the immune system (Nagata, 2018). This prevents leakage of intracellular components into the extracellular space thus reduces inflammation (Elmore, 2007; Yee and Li, 2021) further decreasing the likelihood of side effects if translated to an *in vivo* model.

### 4.4 Does the methanolic extract of *B. carterii* produce cell cycle dysregulation?

Many cancer cells overexpress cell division promoting proteins (Nenclares and Harrington, 2020). As such, another target of chemotherapeutic agents is the cell cycle thus prevention of uncontrolled, indefinite cancer cell division (Hanahan and Weinberg, 2011; Bai et al., 2017; Jhaveri et al., 2021). Previous work suggests that *B. serrata* extracts can induce _sub_G_1_ and G_1_ cell cycle arrests *in vitro* (Ranjbarnejad et al., 2017; Trivedi et al., 2023) though the underlying mechanisms remain unclear. Therefore, we determined whether *B. carterii* oleoresin methanolic extract impacted cell cycle regulation. Figures 5A, B show that extracts increase the proportion of cells in _sub_G_1_ which is indicative of DNA damage (Plesca et al., 2008; Bergandi et al., 2018). Furthermore, the accumulation of cells in G_1_ and decrease of cells in S phase suggests inhibition of transition which ought to prevent, or at least slow division (Bertoli et al., 2013).

To better understand the molecular mechanisms underlying cell cycle dysregulation we measured the expression of key regulatory proteins (Figures 5C, D). *B. carterii* oleoresin methanolic extract did not *significantly* alter the expression of p18; a protein that inhibits transition from G_1_ to S phase of the cell cycle. However, the extract reduced the expression of cyclins D1 and D3, and CDK2 and CDK6; proteins that facilitate the transition from G_1_ to S phase of the cell cycle (Nenclares and Harrington, 2020).

Our data suggests it is the downregulation of transition-promoting proteins, rather than upregulation of inhibitory proteins that is primarily responsible for the proportional changes reported in Figure 5B. However, we cannot rule out a role for other inhibitory proteins such as p21 and p27 (Foster et al., 2010; Bai et al., 2017). For this, further work is required.

### 4.5 The wider therapeutic potential of *B. carterii*

There remains a global need to develop new anti-cancer drugs given the ever present off-target effects and increasing incidence of drug resistance associated with existing compounds. Our data suggests that *B. carterii* may be a promising source of novel anti-cancer agents. The cytotoxic effects were specific and produced by the controlled mechanisms of apoptosis and cell-cycle arrest; common targets for other chemotherapeutics. Furthermore, we know that oleresins from other *Bosweillia* species are well tolerated in animal models and clinical trials (Gupta et al., 1998; Kimmatkar et al., 2003; Sengupta et al., 2008). The therapeutic potential of *B. carterii* may be of value in low-income and developing countries. Here, mainstream chemotherapeutics are frequently inaccessible so plant-based and traditional medicines are used widely (Khan, 2014). However, further research into the pharmacodynamics and pharmacokinetics of *B. carterii* oleoresin methanolic extracts is required.

### 4.6 Summary

We are the first to report that methanolic extracts of *B. carterii* are selectively cytotoxic against three leukemia cell lines. Cytotoxicity results from 1) activation of the intrinsic apoptotic pathway and 2) cell cycle arrest through downregulation of CDK2, CDK6, cyclin D1 and cyclin D3. These data suggest that *B. carterii* may be an important source of potential novel chemotherapeutic drugs. This warrants further investigation to elucidate and purify the precise compounds responsible for the reported effects.

## Data Availability

The raw data supporting the conclusion of this article will be made available by the authors, without undue reservation.
