# Supplementary material for: Boswellia carterii oleoresin extracts induce caspase-mediated apoptosis and G1 cell cycle arrest in human leukaemia subtypes
Source: Front Pharmacol. 2023 Dec 14;14:1282239. doi: 10.3389/fphar.2023.1282239 (PMC10752984; doi:10.3389/fphar.2023.1282239)
Supplement: Supplementary file 1 [file Table1.DOCX]

Supplementary Material

# Supplementary Data

## Extract characterization using high performance liquid chromatography with diode-array detection (HPLC-DAD)

### Sample preparation

Each *B. carterii* solvent extract was prepared to a concentration of 12.5 mg/ml in 99.9 % HPLC grade acetonitrile and syringe filtered through a 0.22 µm syringe filter before injection. All solvent extracts were prepared in an identical manner before high performance liquid chromatography with diode-array detection (HPLC-DAD) analysis. Sample preparation was identically performed for the characterization of all *B. carterii* extracts.

### Gradient high performance liquid chromatography with diode-array detection (HPLC-DAD)

Gradient HPLC-DAD analysis was performed using an Agilent 1260 infinity HPLC system fitted with a 1260 quaternary pump, a 1260 standard infinity autosampler fitted with a 100 µl sample loop, a 1260 Infinity thermostatted column compartment (set to 25 °C) and a 1260 Infinity diode array and multiwavelength detector (All equipment from Agilent Technologies, USA). The HPLC column used for all analyses was an Agilent 5HC C-18(2) 150 x 4.6mm column. Samples were drawn up at a rate of 200 µl/minute to a total injection volume of 20 µl per sample run. HPLC was performed using a gradient method where the concentration of the mobile phase and flow rate was programmed using the software ChemStation (Agilent Technologies, USA) to automatically adjust over time. The gradient HPLC method used for analysis is described in supplementary table S1. HPLC separation was performed for a run time of 60 minutes, with spectra generated at wavelengths of 205, 230, 250, 270, 290 and 310 nm.

**Supplementary Table S1. The gradient high-performance liquid chromatography method used for all characterization of each *B. carterii* oleoresin extracts.**

| **Time (Min)** | **Acetonitrile (%)** | **dH_2_O (%)** | **Flow rate (mL/min)** |
| --- | --- | --- | --- |
| **0** | 50 | 50 | 0.8 |
| **3** | 50 | 50 | 0.8 |
| **6** | 95 | 5 | 1 |
| **15** | 95 | 5 | 1.2 |
| **56** | 95 | 5 | 1.2 |
| **59** | 50 | 50 | 0.8 |

## Antibodies used for Western blotting

Antibodies used for the study were the following: Cleaved Caspase-7 (Asp198) (D6H1) Rabbit mAb, Caspase-7 (D2Q3L) Rabbit mAb, Cleaved Caspase-9 (Asp330) (E5Z7N) Rabbit mAb, Caspase-9 (C9) Mouse mAb, Cleaved PARP (Asp214) (D64E10) XP® Rabbit mAb, PARP Antibody, Caspase-3 (D3R6Y) Rabbit mAb, Cleaved Caspase-3 (Asp175) (5A1E) Rabbit mAb. p53 (7F5) Rabbit mAb, Cyclin D1 (92G2) Rabbit mAb, CDK2 (78B2) Rabbit mAb, p18 INK4C (DCS118) Mouse mAb, CDK6 (DCS83) Mouse mAb, Cyclin D3 (DCS22) Mouse mAb, CDK4 (D9G3E) Rabbit mAb and GAPDH (D16H11).

The secondary antibodies used were the following: anti-mouse IgG, Horseradish peroxidase (HRP)-conjugated antibody and anti-rabbit IgG, HRP-conjugated antibody (All purchased from Cell Signalling Technologies, USA).

# Supplementary Figures and Tables

## Supplementary Figures

**Supplementary Figure S1. Gradient high performance liquid chromatography with diode-array detection characterization of the acetonitrile *B. carterii* oleoresin solvent extract at variable wavelengths (205-310nm).**


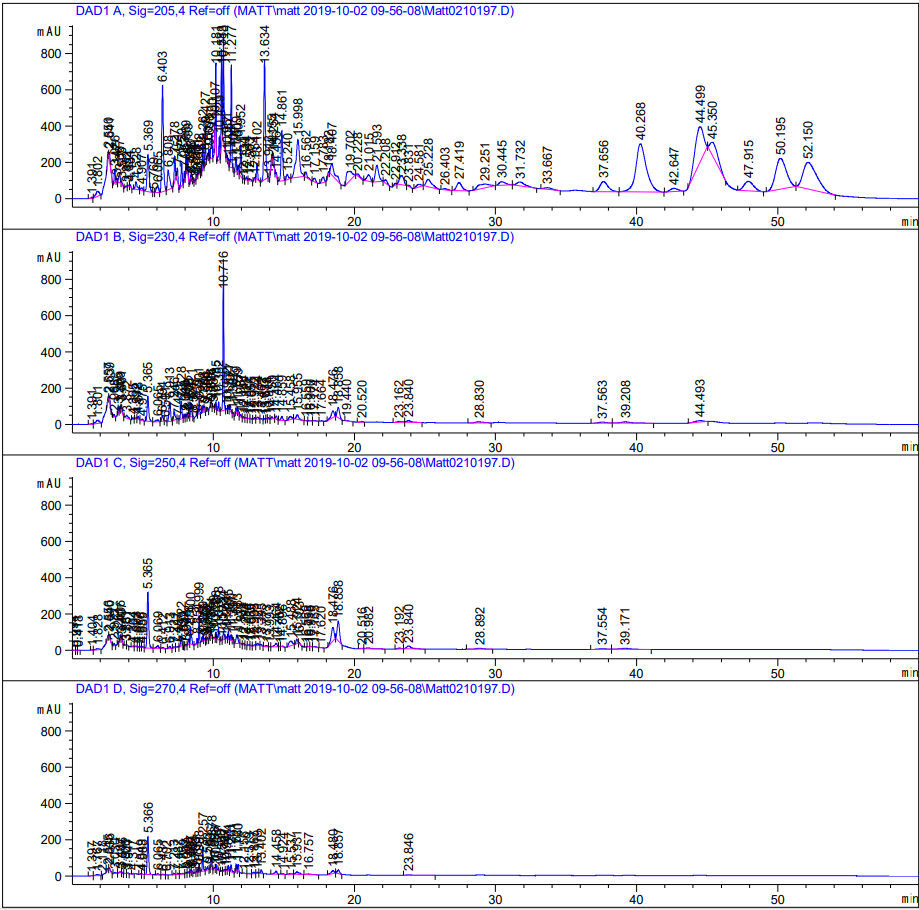

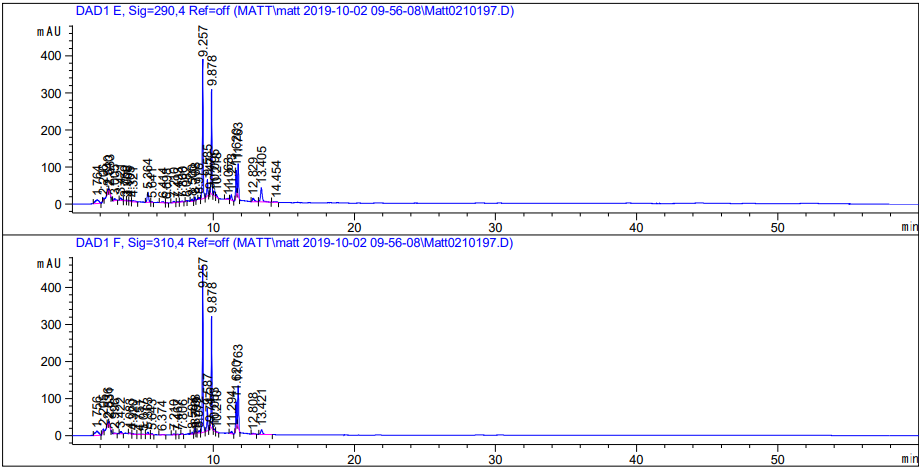


**205nm**

**230nm**

**250nm**

**270nm**

**290nm**

**310nm**

**Supplementary Figure S2. Gradient high performance liquid chromatography with diode-array detection characterization of the ethanol *B. carterii* oleoresin solvent extract at variable wavelengths (205-310nm).**


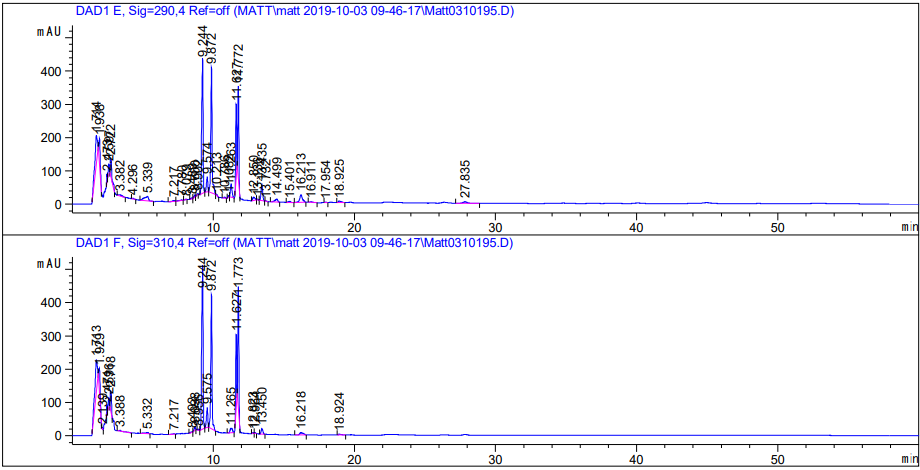

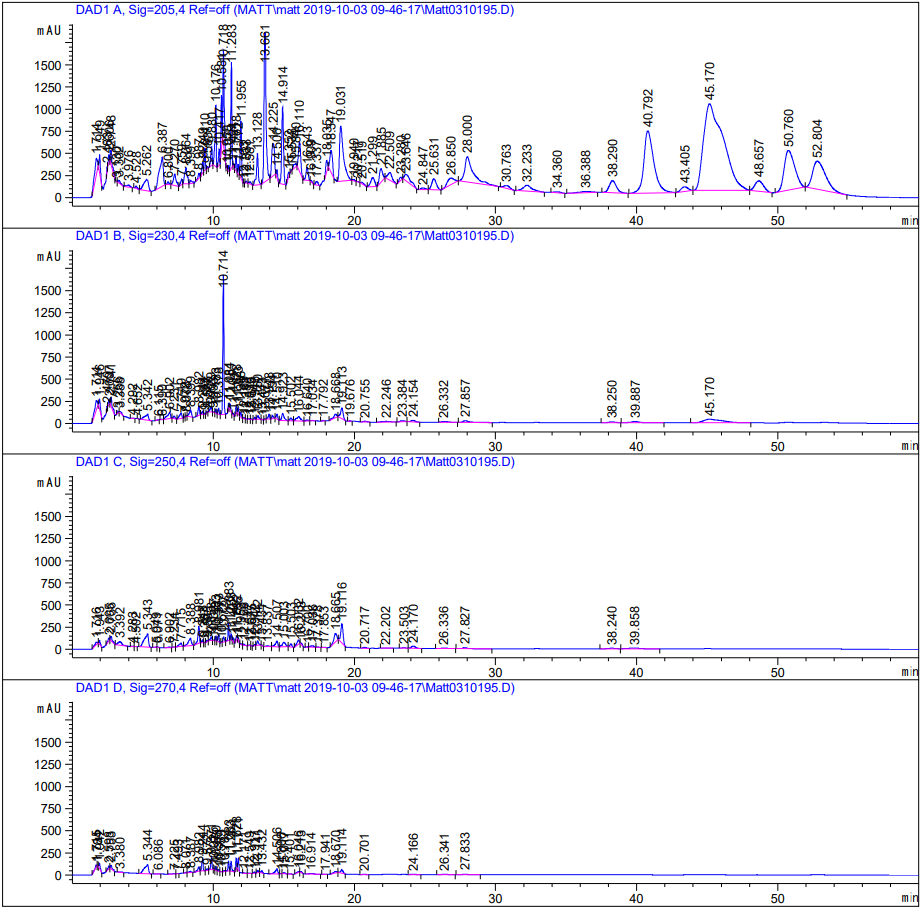


**205nm**

**230nm**

**250nm**

**270nm**

**290nm**

**310nm**

**Supplementary Figure S3. Gradient high performance liquid chromatography with diode-array detection characterization of the methanol *B. carterii* oleoresin solvent extract at variable wavelengths (205-310nm).**


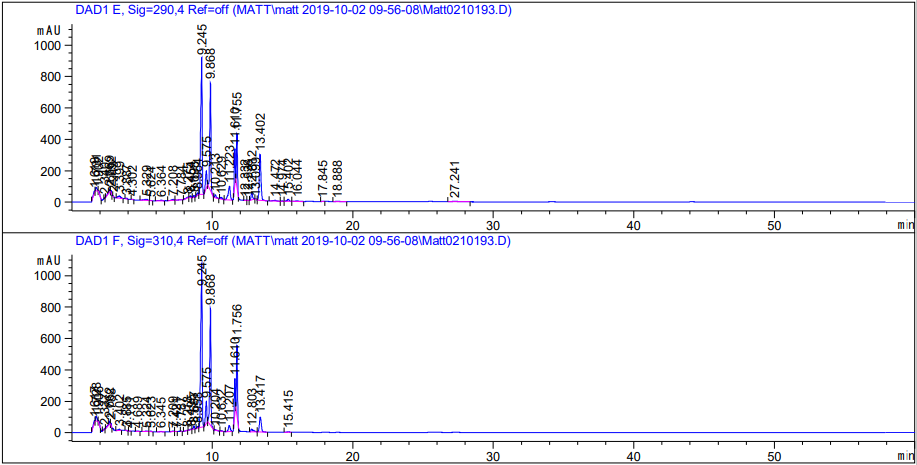

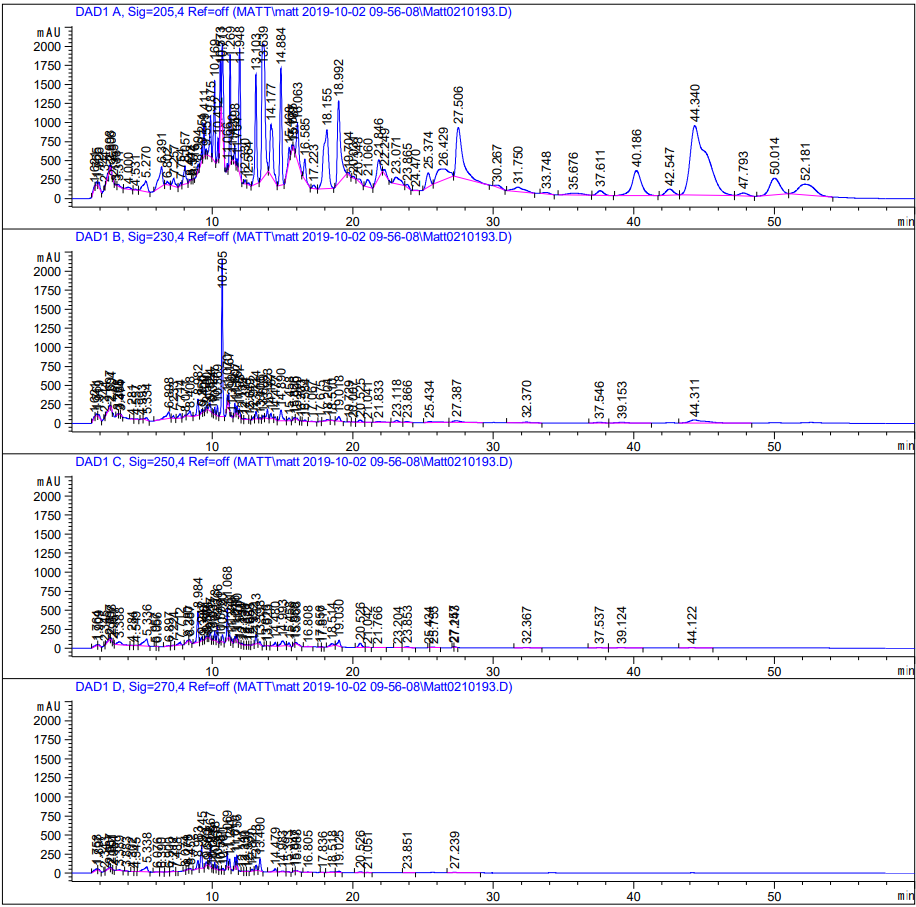


**205nm**

**230nm**

**250nm**

**270nm**

**290nm**

**310nm**

**Supplementary Figure S4. Gradient high performance liquid chromatography with diode-array detection characterization of the propan-2-ol *B. carterii* oleoresin solvent extract at variable wavelengths (205-310nm).**


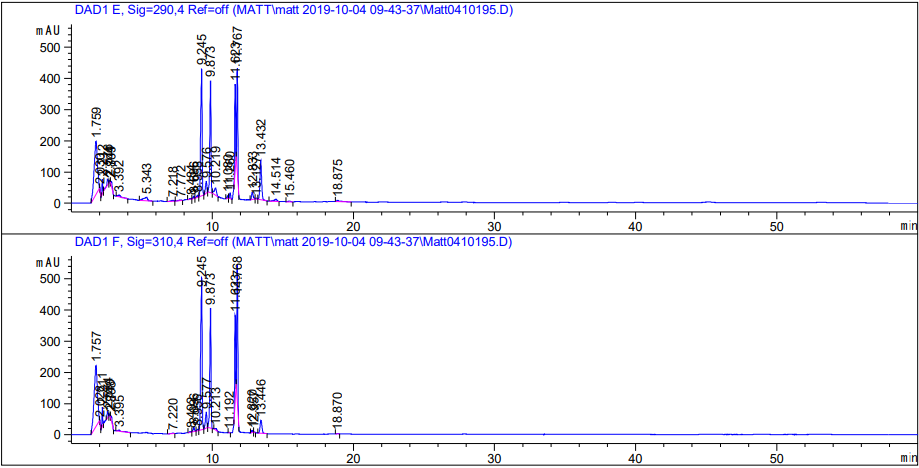

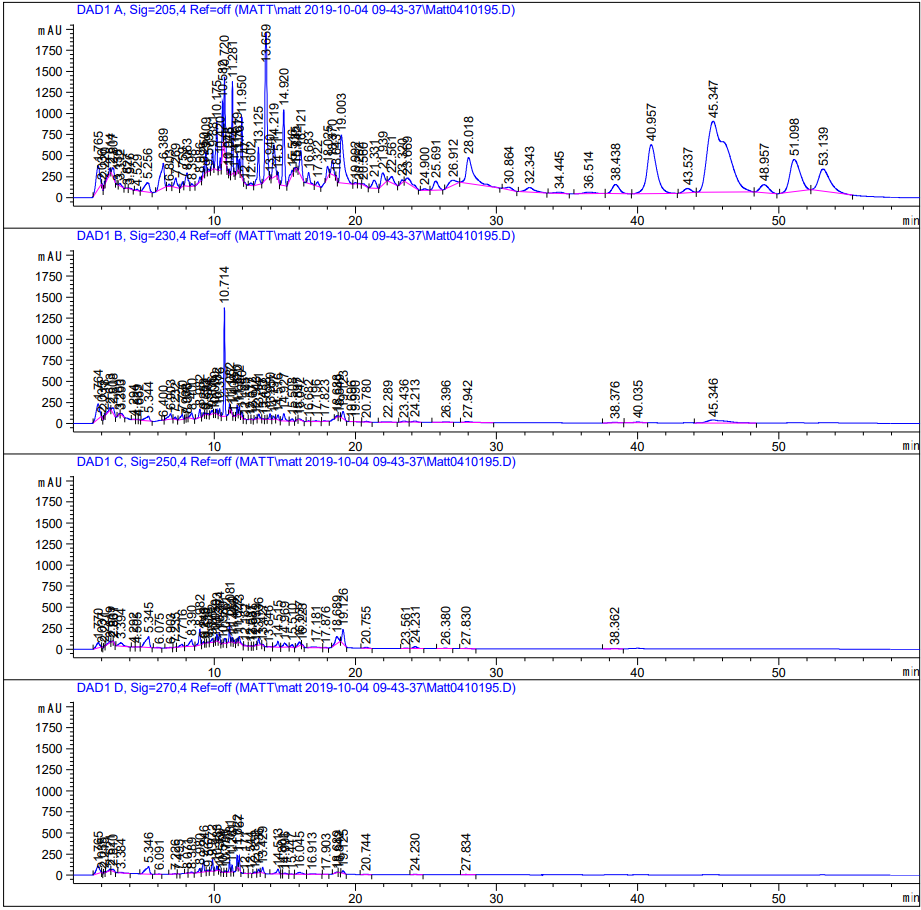


**205nm**

**230nm**

**250nm**

**270nm**

**290nm**

**310nm**


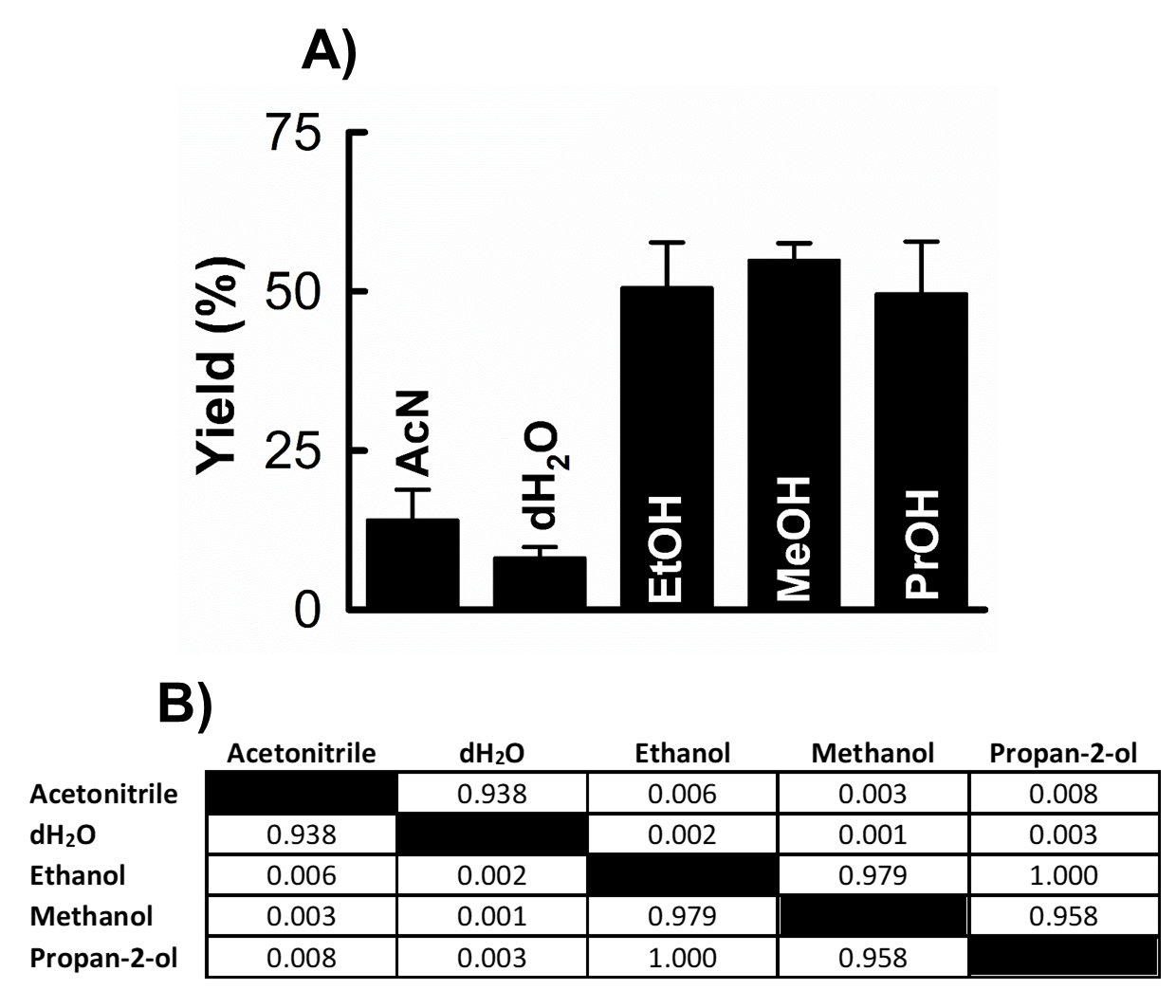
**Supplementary Figure S5.** **The effect of solvent on *B. carterii* extraction yield. (A)** The extraction yield following extraction of *B. carterii* oleoresin with acetonitrile, distilled water, ethanol, methanol and propan-2-ol. Statistical indicators have been removed for clarity, see S1B for statistical analysis **(B)** The p values following a Tukey post-hoc test pairwise comparisons on the extraction yields recovered using each extraction solvent. Statistical significant difference indicated by a p value < 0.05.

**Supplementary Figure S6.** **The *B. carterii* oleoresin methanolic extract induces**
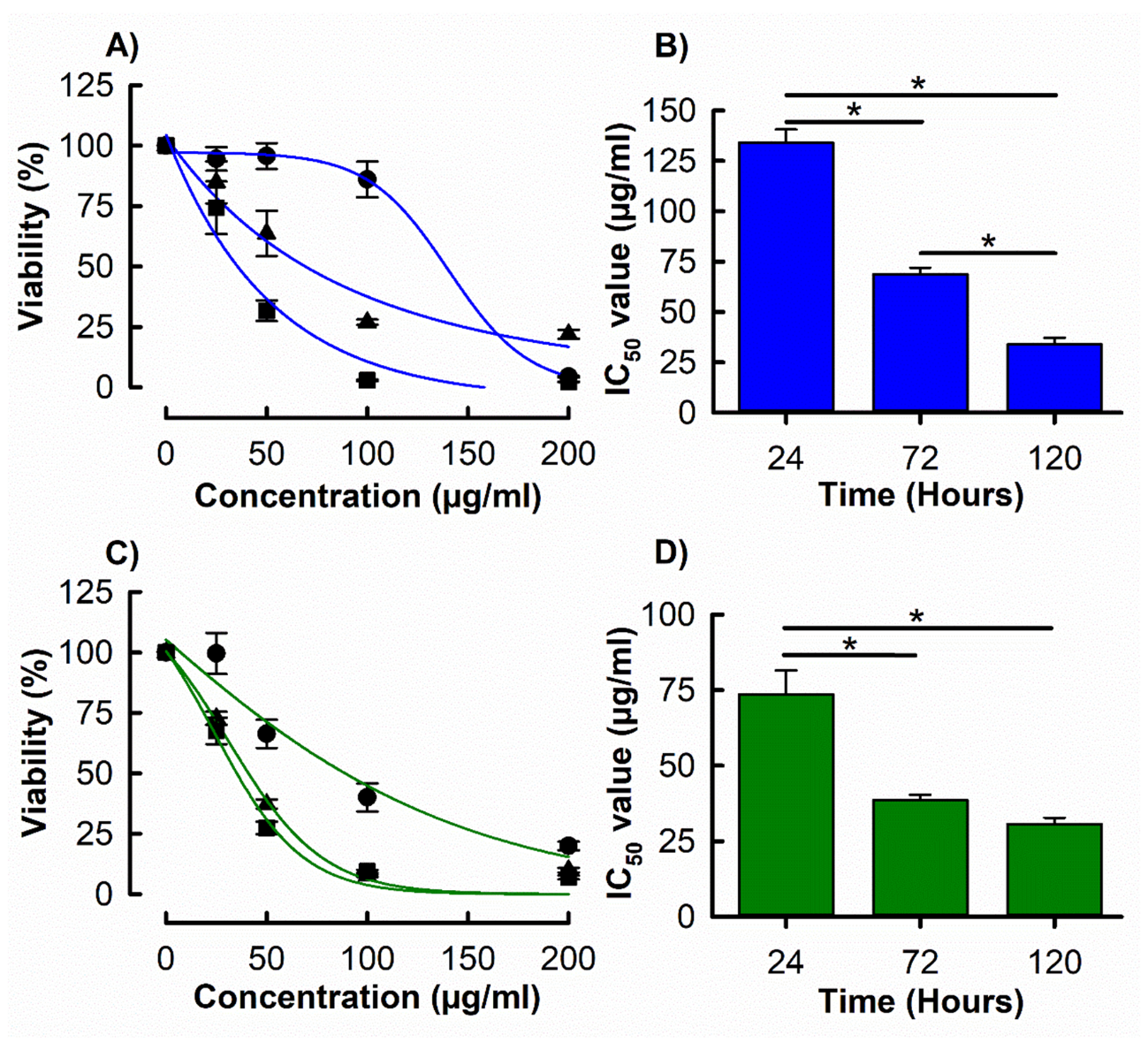
 **time dependent cytotoxicity against MOLT-4 and CCRF-CEM cells. (A)** Dose response curves comparing the cytotoxicity of the *B. carterii* oleoresin methanolic extract against MOLT-4 cells following treatment for 24 (●), 72 (▲) and 120 (■) hours. All data points are normalized with vehicle alone (0.5 % *v/v* DMSO). **(B)** Corresponding normalised mean ± SEM IC_50_ values from 12 repeats. **(C)** Dose response curves comparing the cytotoxicity of the *B. carterii* oleoresin methanolic extract against CCRF-CEM cells following treatment for 24 (●), 72 (▲) and 120 (■) hours. All data points are normalized with vehicle alone (0.5 % *v/v* DMSO). **(D)** Corresponding normalised mean ± SEM IC_50_ values from 12 repeats. Statistically significant (p <0.05) pairwise comparisons are indicated by asterixis (*).
